# An Overview on Synthetic 2-Aminothiazole-Based Compounds Associated with Four Biological Activities

**DOI:** 10.3390/molecules26051449

**Published:** 2021-03-07

**Authors:** Mohamed Farouk Elsadek, Badreldin Mohamed Ahmed, Mohamed Fawzi Farahat

**Affiliations:** 1 Department of Community Health Sciences, College of Applied Medical Sciences, King Saud University, P.O. Box 10219, Riyadh 11433, Saudi Arabia; bmohamed@ksu.edu.sa (B.M.A.); mffarahat@ksu.edu.sa (M.F.F.); 2 Nutrition and Food Science Department, Faculty of Home Economics, Helwan University, P.O. Box 11795, Cairo 11511, Egypt

**Keywords:** 2-aminothiazoles, antibacterial, anti-inflammatory

## Abstract

Amongst sulfur- and nitrogen-containing heterocyclic compounds, the 2-aminothiazole scaffold is one of the characteristic structures in drug development as this essential revelation has several biological activities abiding it to act as an anticancer, antioxidant, antimicrobial and anti-inflammatory agent, among other things. Additionally, various 2-aminothiazole-based derivatives as medical drugs have been broadly used to remedy different kinds of diseases with high therapeutic influence, which has led to their wide innovations. Owing to their wide scale of biological activities, their structural variations have produced attention amongst medicinal chemists. The present review highlights the recently synthesized 2-aminothiazole-containing compounds in the last thirteen years (2008–2020). The originality of this proposal is based on the synthetic strategies developed to access the novel 2-aminothiazole derivatives (*N*-substituted, 3-substituted, 4-substituted, multi-substituted, aryl/alkyl substituents or acyl/other substituents). The literature reports many synthetic pathways of these 2-aminothiazoles associated with four different biological activities (anticancer, antioxidant, antimicrobial and anti-inflammatory activities). It is wished that this review will be accommodating for new views in the expedition for rationalistic designs of 2-aminothiazole-based medical synthetic pathways.

## 1. Introduction

Heterocyclic compounds are so important due to their versatile applications. A large number of heterocyclic compounds containing nitrogen and sulfur are used as medicine in different therapeutic targets. Thiazole is one of the important pharmacophores in drug discovery and development processes. There are many substituted thiazole-containing heterocycles covering a wide range of therapeutic targets including antimicrobial, anticancer, anti-inflammatory and anti-HIV. Aminothiazole scaffolds are important structural units in medicinal chemistry as they have shown antitumor [1,2,3], antiviral [4,5,6], antibacterial [7,8,9], anti-prion [10], psychotropic [11], anti-allergic [12], anti-hypertensive [13], anti-inflammatory [14,15], antifungal [16], antitubercular [17,18], anti-HIV [19], pesticidal [20], antiprotozoal [21], antipyretic [22], antioxidative [23] and analgesic activities [24]. Aminothiazole compounds act as ligands of estrogen receptors [25] and afford a new group of adenosine receptor antagonists [26]. They are also utilized as fungicides, inhibiting the in vivo growth of *Xanthomonas*, or as schistosomicidal and anthelmintic drugs [27].

## 2. Results

### 2.1. 2-Aminothiazoles as Anticancer Agents

**Scheme 1:**
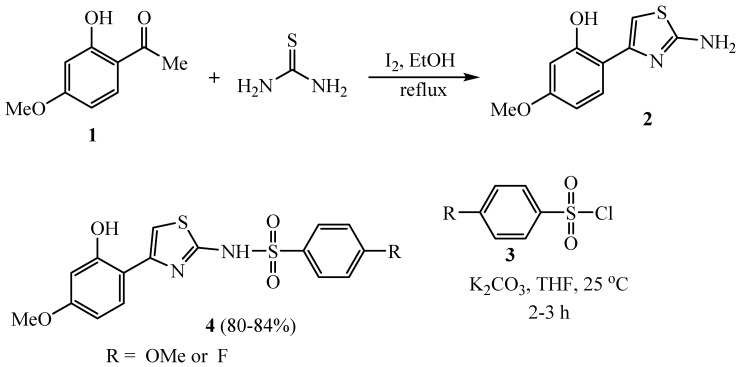
Synthesis of various paeonol-2-aminothiazole-phenylsulfonyl derivatives **4.**

Clinical administration of high doses of anticancer drugs to defeat resistance leads to severe toxicities [28]. The literature survey revealed that heterocyclic thiazole derivatives were integrated with other moieties to evaluate their anticancer effect. The synthetic protocol of paeonol-2-aminothiazole-phenylsulfonyl derivatives **4** involved treating paeonol **(1)** with thiourea and iodine in refluxing ethyl alcohol to furnish the corresponding 2-aminothiazole scaffold **2**, which was treated with phenylsulfonyl chloride **3** that had been substituted to produce the final wanted compound **4** (Scheme 1). The cytotoxic effects of various paeonol-2-aminothiazole-phenylsulfonyl derivatives **4** were assessed against fibroblast cells (BALB/3T3) and seven cancer cell lines. The F and OCH_3_ derivatives of the thiazole-paeonolphenylsulfonyl scaffold showed cytotoxic potent effects against the tested cancer cell lines [29].

**Scheme 2:**
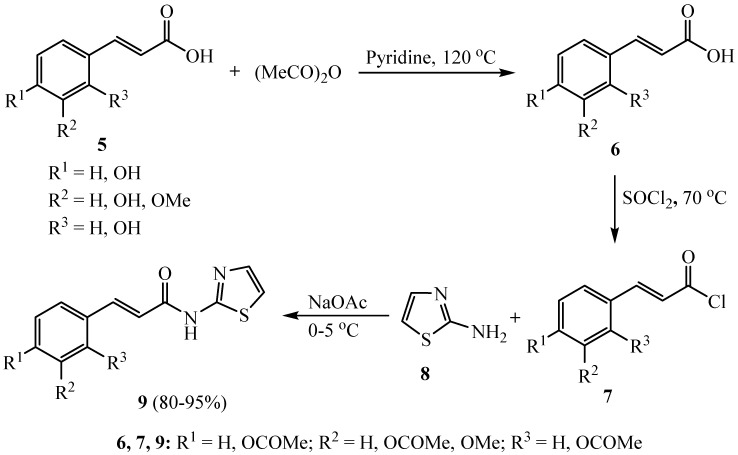
Synthesis of cinnamic acid amide scaffolds **9.**

New cinnamic acid amide scaffolds **9** comprising thiazoles were designed and synthesized as outlined in Scheme 2. The results of anticancer activity of this work indicated that compound **9** (R^1^ = R^2^ = H, R^3^ = OCOMe) features potential characteristics for drug development combining both coagulant and platelet effects [30].

**Scheme 3:**
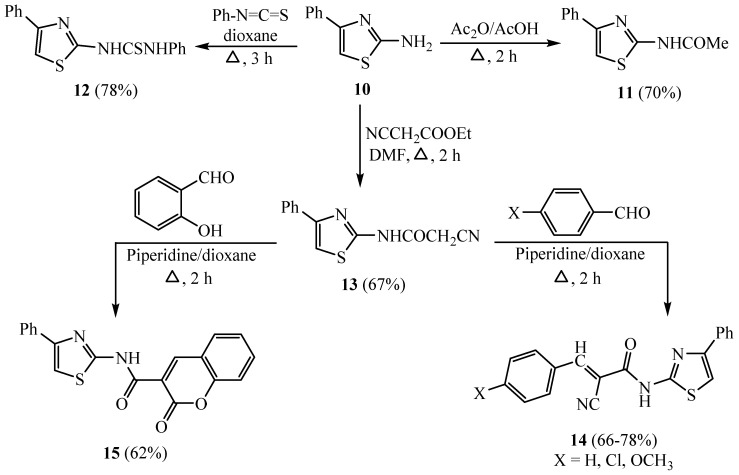
Synthesis of coumarin derivative **15.**

The thiazole derivative **10** was produced by heating phenacyl bromide with thiourea in ethyl alcohol, which acetylated by acetic anhydride to furnish the corresponding *N*-acetyl compound **11**. The nucleophilic addition of **10** to phenyl isothiocyanate afforded the *N*-phenylthiourea derivative **12**. In contrast, the reaction of **10** with ethyl cyanoacetate in dimethyl formamide produced *N*-cyanoacetamide derivative **13**. Condensation of **13** with three types of substituted benzaldehydes (namely, benzaldehyde 4-chlorobenzaldehyde or 4-methoxybenzaldehyde) produced the corresponding benzylidene derivatives **14**. In addition, when compound **13** reacted with salicylaldehyde, it gave the coumarin derivative **15** (Scheme 3) [31].

**Scheme 4:**
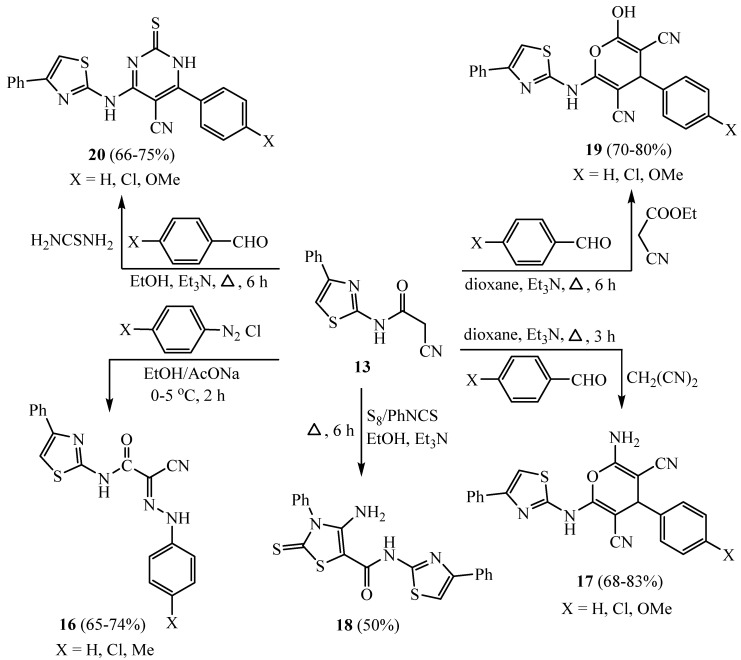
Synthesis of pyran derivatives **19a–c.**

The reaction of acetamide derivative **13** with various aryl diazonium chlorides affords the aryl hydrazone compounds **16**. In addition, the multi-component reaction of **13** with substituted benzaldehydes and malononitrile produced the pyran derivatives **17** (Scheme 4). Compound **13** was applied for thiazole synthesis, and as a result, compound **13** reacted with elemental sulfur and phenyl isothiocyanate to afford the thiazole derivative **18**. Similarly, the multi-component reaction of **13** with substituted benzaldehydes and ethyl cyanoacetate produced the pyran derivatives **19a**–**c** (Scheme 4). Furthermore, the multi-component reaction of **13** with substituted benzaldehydes and thiourea produced the pyrimidine scaffolds **20**. 2-Amino-4-(4-chlorophenyl)-6-(4-phenylthiazol-2-yl)-4*H*-pyran-3,5-dicarbonitrile (**17**, X = Cl) indicated the maximum cytotoxicity among the synthesized compounds towards six cancer cell lines [31].

**Scheme 5:**
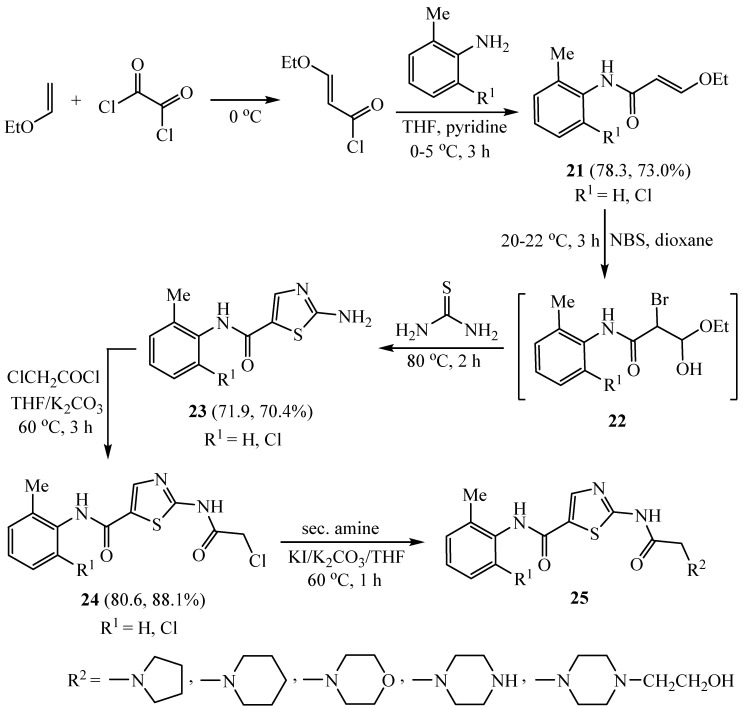
Synthesis of 2-amino-thiazole-5-carboxylic acid phenylamide derivatives.

Treatment of 3-ethoxyacryloyl chloride with either 2-methylaniline or 2-chloro-6-methylaniline in tetrahydrofuran utilizing basic pyridine as a catalyst gave the substituted 3-ethoxyacrylamides **21**, and then treatment of **21** with *N*-bromosuccinimide produced the crude α-formyl-α-bromoacetate hemiacetals **22**. Addition of thiourea to hemiacetals **22** gave the 2-amino-thiazole-5-carboxylic acid phenylamides **23**, which reacted with chloroacetyl chloride in the presence of K_2_CO_3_ as a base to afford the key intermediates **24**. Finally, chloroacetamide derivatives **24** reacted with various secondary amine compounds to afford the final products **25** (Scheme 5). The synthesized series of 2-amino-thiazole-5-carboxylic acid phenylamide derivatives showed good anti-proliferative effects on human K563 leukemia cells [32].

**Scheme 6:**
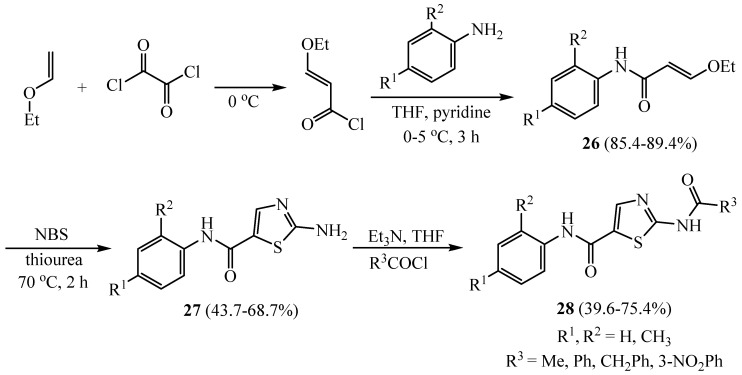
Synthesis of 2-aminothiazole-5-carbamides **28.**

According to the significance of the carboxanilide side chain on the fifth position of the thiazole ring and the cytostatic impact on human chronic myeloid leukemia cell line K562, 2-aminothiazole-5-carbamides **28** were synthesized as outlined in Scheme 6. 3-Ethoxy-*N*-arylpropenamides **26** were synthesized by nucleophilic substitution of suitable aniline onto 3-ethoxyacryloyl chloride. Reaction of the advanced enones **26** with *N*-bromosuccinimide (NBS) followed by a coupling of thiourea installed thiazole ring **27**. The last step was a nucleophilic displacement of acetic anhydride or various benzoyl chlorides to give the corresponding target **28** derivatives [33].

**Scheme 7:**
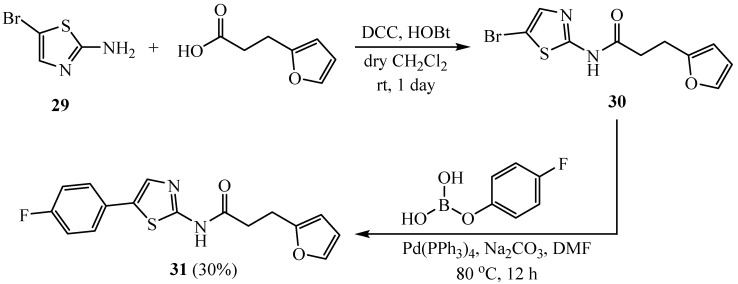
Synthesis of N-(5-(4-fluorophenyl)thiazol-2-yl)-3-(furan-2-yl) propanamide.

The synthesis of *N*-(5-(4-fluorophenyl)thiazol-2-yl)-3-(furan-2-yl)propanamide **(31)** as outlined in Scheme 7 involves the reaction of 2-amino-5-bromothiazole **(29)** with 3-(furan-2-yl)propanoic acid followed by the Suzuki reaction of the amide product **30** with 4-fluorophenylboric acid. Compound **31** affords the potent inhibitory effect on KPNB1 and anticancer activity in cell-based assays [34].

**Scheme 8:**
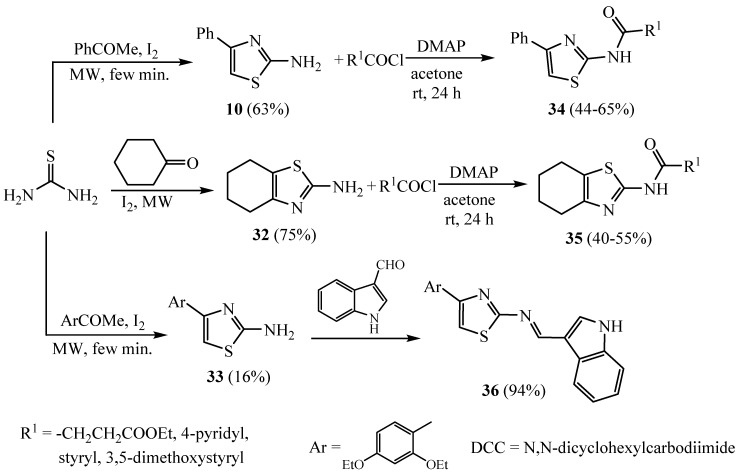
Synthesis of 4,5-substituted-2-aminothiazoles **32–36.**

The synthesis of 4,5-substituted-2-aminothiazoles **10** and **32** (Scheme 8) has been achieved according to the literature by the reaction of acetophenone or cyclohexanone with thiourea in the presence of iodine. Compound **10** or **32** was stirred with acid and/or acyl chloride to afford the corresponding amide compounds **34** (R^1^ = -CH_2_CH_2_COOEt, 4-pyridyl, styryl and 3,5-dimethoxystyryl) and **35** (R^1^ = -CH_2_CH_2_COOEt and 3,5-dimethoxystyryl), respectively. Compound 4-(2,4-diethoxyphenyl)thiazol-2-amine **(33)** was reacted with 1*H*-indole-3- carboxaldehyde in ethyl alcohol to afford the Schiff base compound **36** [35]. Derivatives **32–36** were exhibited as potent Poly(ADP-Ribose) Polymerase-1 inhibitors.

**Scheme 9:**
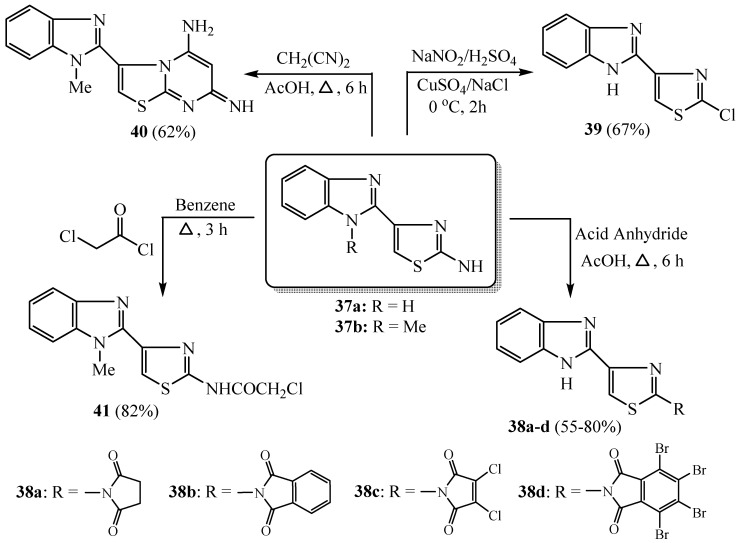
Synthesis of derivatives **38–41.**

Benzimidazole-thiazole derivatives were prepared by heating a mixture of equimolar amounts of 2-acetylbenzimidazoles and thiourea in ethyl alcohol and an excess amount of iodine [36]. The acid anhydride effect on compound **37a** was studied (Scheme 9) which was condensed with various acid anhydrides (namely, succinic anhydride, phthalic anhydride, dichloromalic anhydride and/or tetrabromophthalic anhydride) in acetic acid to produce the wanted anhydride compounds **38a–d** [37]. It is known that an aromatic amino group substitution is workable by its diazonium salt preparation and subsequent replacement with a nucleophile via Sandmeyer reactions. Therefore, compound **37a** was reacted with CuSO_4_, NaNO_2_ and NaCl to afford the corresponding 2-chlorothiazole scaffold **39**. Meanwhile, **37a** was condensed via different acid anhydrides to give the corresponding derivatives **38a–d**. In contrast, compound **37b** was reacted with malononitrile in acetic acid to produce 5-amino-pyrimidine derivative **40** [37]. The compound **37b** was acylated with chloroacetyl chloride to give the corresponding chloroacetamide compound **41** (Scheme 9). All of these synthesized derivatives showed respectable anticancer activities toward HepG2 and PC12 cell lines.

**Scheme 10:**
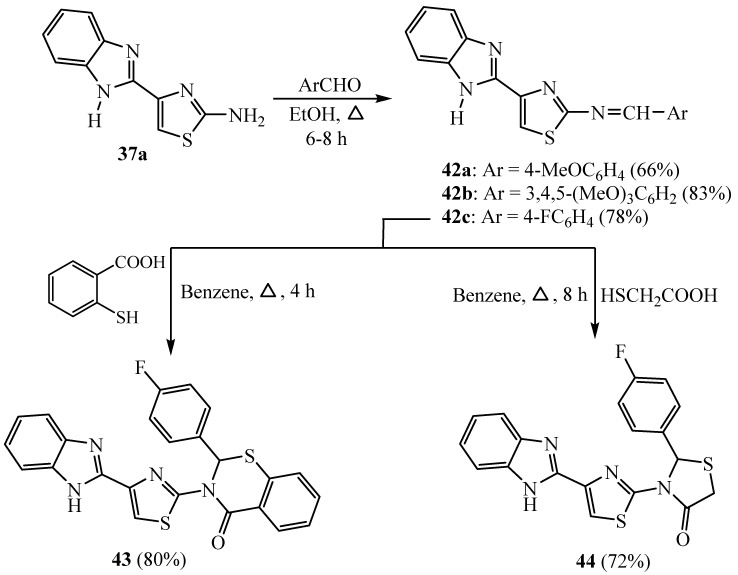
Synthesis of derivatives **42–44.**

Meanwhile, Schiff’s bases are deemed significant intermediates for the synthesis of other heterocycles. Consequently, Schiff’s bases **42a–c** were constructed by the reaction of **37a** with different substituted benzaldehydes, namely, 4-methoxybenzaldehyde, 3,4,5-trimethoxybezaldehye and/or 4-fluorobenzaldehyde in ethanol to produce *N*-(substituted)-thiazol-2-amine **42a–c,** respectively. A set of compounds comprising thiazolidinone and benzothiazine nuclei were accomplished by cyclizing Schiff’s base **42c** by either thiosalicylic acid and/or thioglycolic acid to get thiazolidinone and benzothiazinone **43** and **44** derivatives, respectively [37] (Scheme 10). Meanwhile, compounds **43** showed promising anticancer activity against both of HepG2 and PC12 cell lines.

**Scheme 11:**
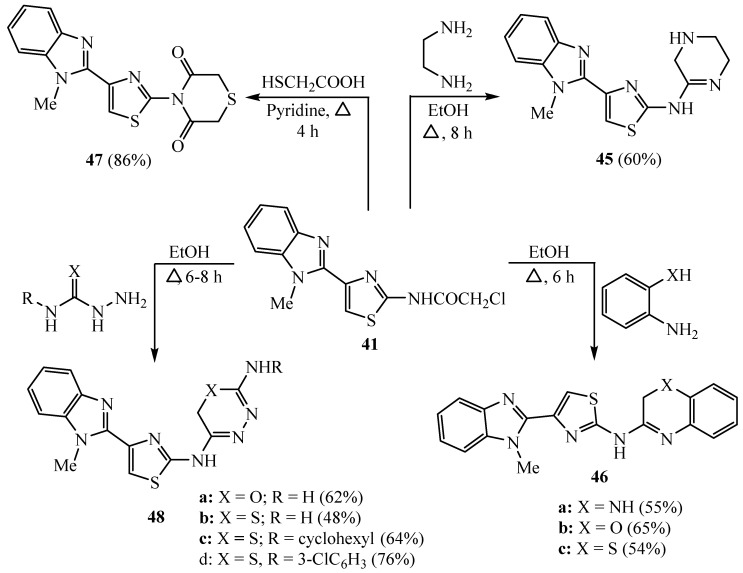
Synthesis of derivatives **45–48.**

Additionally, aminothiazole derivative **41** was cyclized with 1,2-ethylenediamine and/or ortho-substituted aniline compounds to give the corresponding 2-amino-pyrazine **45** and **46a–c**, respectively. In contrast, compound **41** was cyclized with HS-CH_2_-COOH to produce thiazinedione scaffold **47**. Furthermore, compound **41** was cyclized with various semicarbazide or thiosemicarbazide derivatives to afford 1,3,4-oxadiazine **48a** and 1,3,4-thiadiazine **48b–d** compounds [37] (Scheme 11). Among these derivatives, **48c** showed high activity against the PC12 anticancer cell line.

**Scheme 12:**
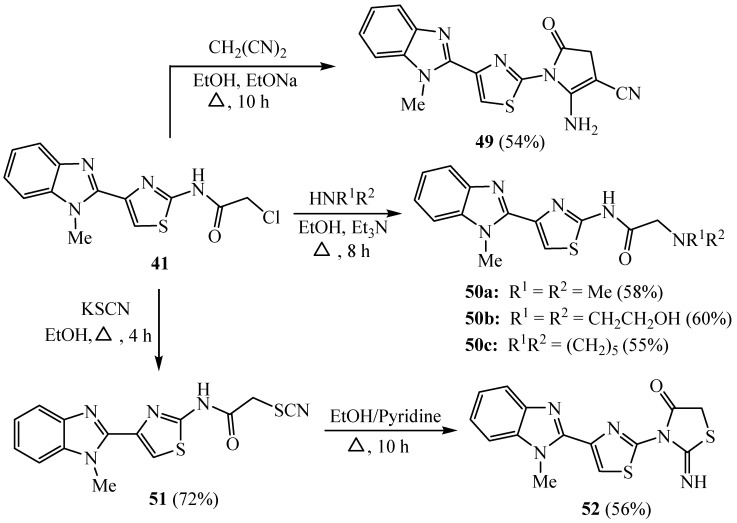
Synthesis of derivatives **49–52.**

Compound **41** containing an aminothiazole moiety was heated with malononitrile in sodium ethoxide to give the corresponding 3-cyano-5-oxo-1*H*-pyrrole derivative **49**. In addition, treatment of compound **41** with different secondary amines gave the acetamide derivatives **50a–c**. Moreover, treatment of **41** with potassium thiocyanate produced the thiocyanate-acetamide scaffold **51**, which was cyclized to yield the corresponding thiazolidinone **52** (Scheme 12). The prepared compounds have potent anticancer activity against PC12 and HepG2 cell lines [37].

**Scheme 13:**
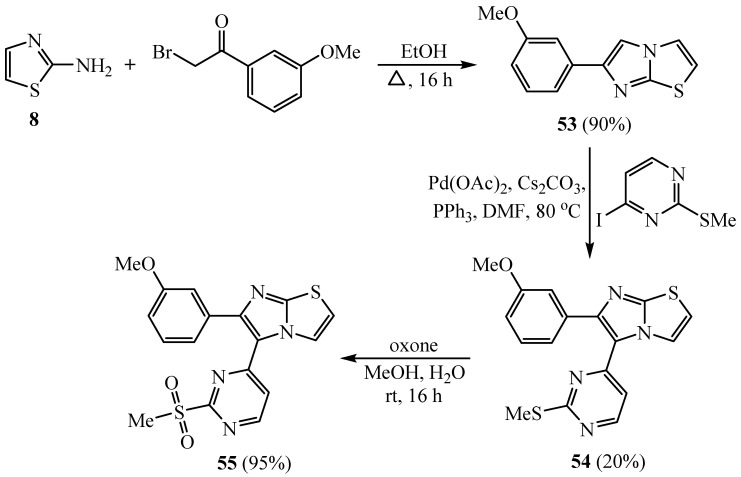
Synthesis of derivatives **53–55.**

Heterocyclization of 2-aminothiazole **8** with α-bromo-3-methoxyacetophenone proceeded by heating in ethyl alcohol to yield 6-(3-methoxyphenyl)imidazo[2,1-*b*]thiazole **53**, which underwent heating with 4-iodo-2-(methylthio)pyrimidine in the presence of palladium acetate, cesium carbonate and triphenyl phosphine to give the corresponding methyl thiopyrimidinyl compound **54** (Scheme 13). The sulfide moiety of **54** was oxidized by oxone to produce the corresponding sulfonyl compound **55** [38]. The derivatives **53–55** were utilized as precursors for the synthesis of compounds **58–61** that displayed a remarkable activity toward the A375P human melanoma cell line (HepG2).

**Scheme 14:**
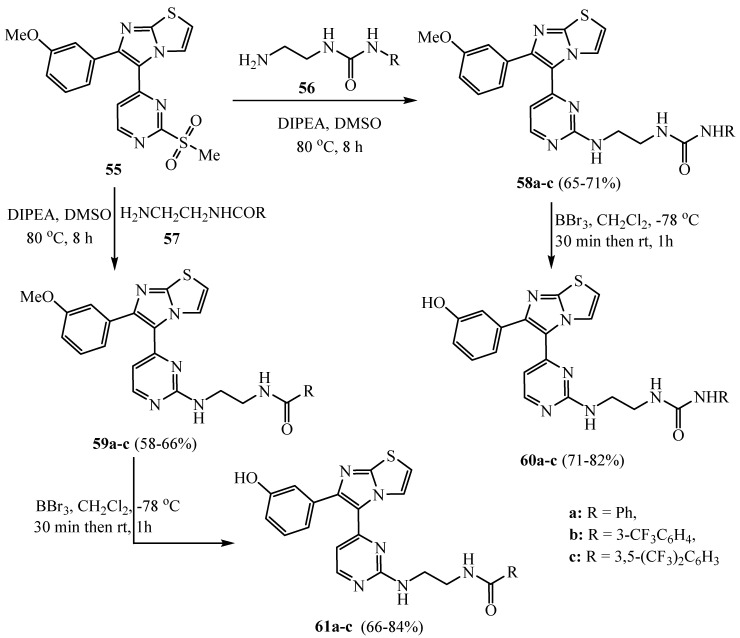
Synthesis of derivatives **58a–c–61a–c.**

Heating of the sulfone-containing thiazolyl moiety compound **55** with the urea and/or amide reagents **56** and **57** in the presence of DIPEA (*N*,*N*-Diisopropyl ethyl amine) gave the target methoxy compounds **58** and **59**, respectively. Demethylation of the methoxy group of **58** and **59** using boron tribromide produced the corresponding hydroxyl target compounds **60a–c** and **61a–c**, respectively (Scheme 14) [38]. The prepared derivatives **58b**, **58c**, **60b**, **59b**, **61a** and **61b** showed superior potency against the A375P “human melanoma cell line” than sorafenib. Moreover, derivatives **61a** and **61b** revealed the highest potency (IC50 = 0.5 and 2.1 µM, respectively). Derivatives with *m*-hydroxyphenyl on the imidazothiazole moiety such as **60b**, **61a** and **61b** showed greater potency than the parallel methoxy hybrids **58b**, **59a** and **59b**, which may due to the expected hydrogen bond with the hydroxyl group of the receptor site.

**Scheme 15:**
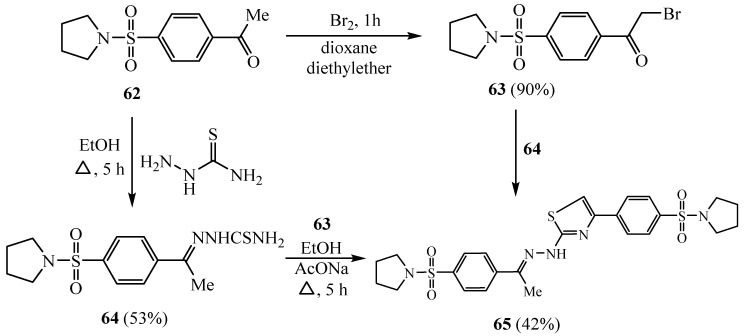
Synthesis of derivatives **63–65.**

Bashandy reported on the preparation of benzenesulfonamide-based heterocycles with expected anticancer activity [39]. Thus, acetophenone derivative **62** was treated with bromine in a mixture of dioxane/diethyl ether to give the alpha bromoacetyl compound **63** (Scheme 15). Treatment of **62** with thiosemicarbazide yielded the thiosemicarbazone moiety **64**, which, when heated with phenacyl bromide compound **63,** produced the thiazole derivative **65**.

**Scheme 16:**
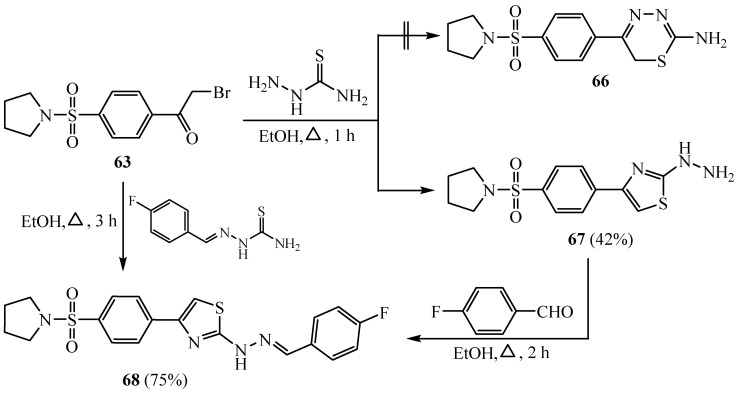
Synthesis of derivatives **67** and **68.**

The interaction of phenacyl bromide compound **63** with thiosemicarbazide furnished the 2-hydrazinyl thiazole compound **67**, instead of 2-aminothiadiazine derivative **66**. Condensation of **67** with 4-fluorobenzyldehyde afforded the corresponding thiazolyl Schiff base **68** which displayed good activity towards hepatocellular carcinoma [39] (Scheme 16).

**Scheme 17:**
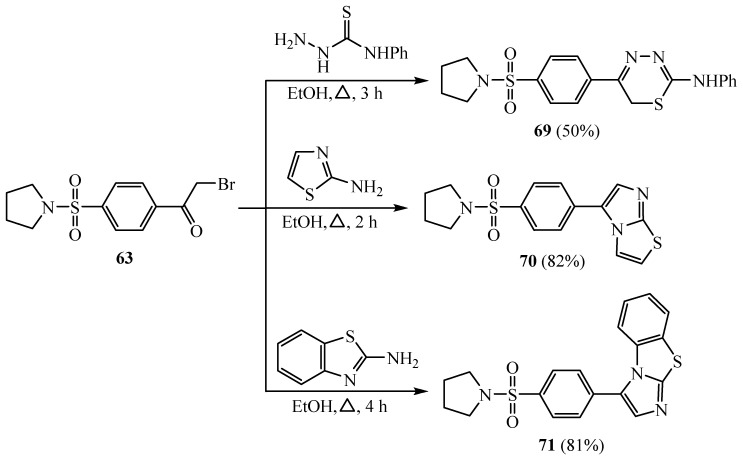
Synthesis of derivatives **69–71.**

Cyclocondensation of **63** with phenyl thiosemicarbazide gave thiadiazine derivative **69**. When compound **63** was treated with 2-aminothiazole and 2-aminobenzothiazole in hot ethyl alcohol, it gave the corresponding imidazo[2,1-*b*]thiazole derivatives **70** and **71**, respectively (Scheme 17) [39]. Derivatives **69** and **71** presented a potent cytotoxicity against both the human liver hepatocellular carcinoma cell line (HepG2) and mammalian cells of the African green monkey kidney cell line (VERO).

Meanwhile, derivative **70** showed good results in relation to the selectivity index (SI), which is the ratio of the concentration that causes 50% death in the African green monkey kidney (VERO) (CC50) compared to the concentration that causes 50% death in the human liver hepatocellular carcinoma cell line (HepG2) (IC50).

**Scheme 18:**
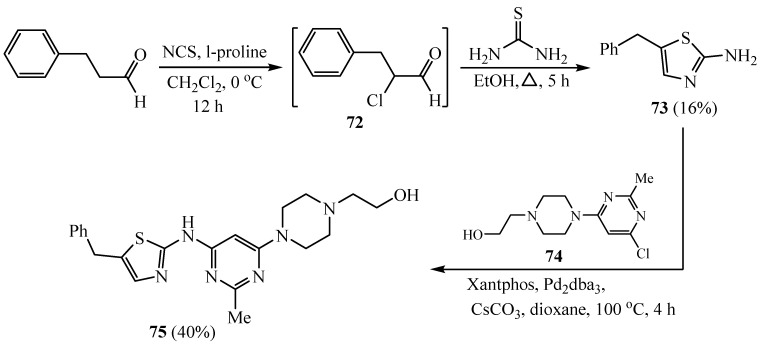
Synthesis of derivatives **73** and **75.**

A potent inhibitor of Src family kinases (SFKs) with slow dissociation rates, aminothiazole derivative **75** of dasatinib, in which the methylene unit substitutes the amide linker between the moiety of thiazole and the aromatic ring of dasatinib, was prepared. The phenyl acetaldehyde was firstly converted to 2-amino5-benzylthiazole **73**, which was combined with **74**, a type of Buchwald reaction (Scheme 18) [40].

**Scheme 19:**
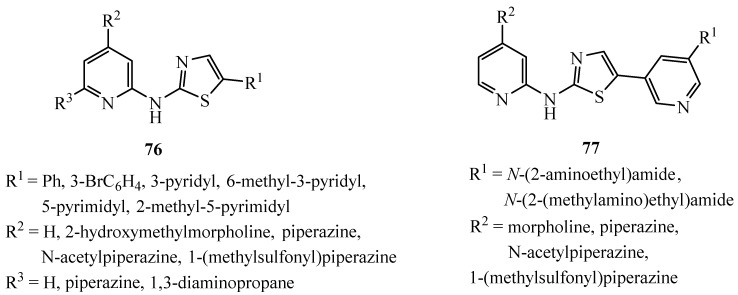
Modification of derivatives **76** and **77.**

A series of 2-pyridylamino-thiazoles and 2-pyridylamino,5-pyridylthiazoles contains a novel category of ATP-competitive Chk1 inhibitors with outstanding inhibitory potential (Scheme 19) [41,42,43]. Modifications of the core with various amides accommodate compound **77** with picomolar potency and very high residence times.

**Scheme 20:**
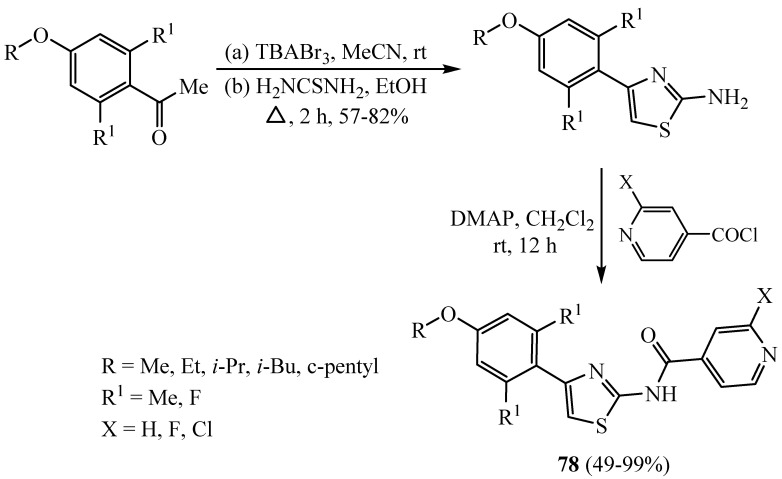
Synthesis of derivative **78.**

Some of acylated 4-aryl-*N*-arylcarbonyl-2-aminothiazole scaffolds **78** were designed and synthesized as highly active Hec1/Nek2 inhibitors. The fluoride derivative of **78** (Scheme 20) pointed to selectivity toward cancer cells over normal phenotype cells and was inactive in a [^3^H]astemizole rival binding assay for hERG liability screening. Thus, 2-aminothiazoles **78** (X = F, R = R^1^ = Me) are promising towards the discovery of a preclinical candidate targeting Hec1/Nek2 [44].

**Scheme 21:**
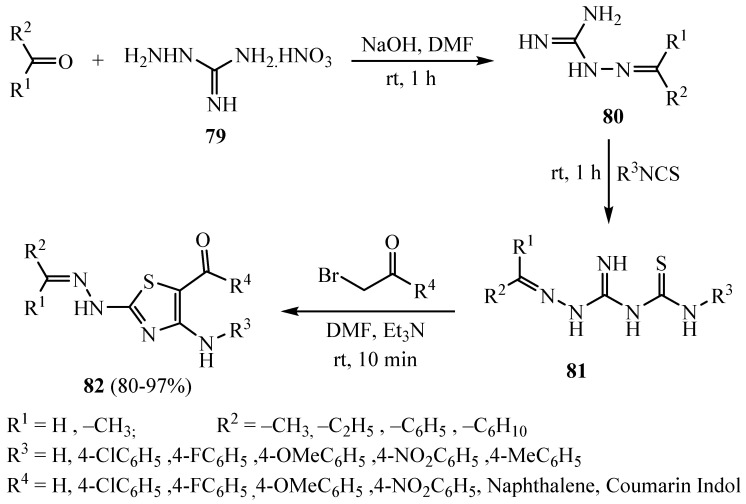
Synthesis of derivative **82.**

The variety elements were incorporated through azomethine linkage on the C4 hydrazine terminus in 2-arylaminothiazoles **82** using (CH_3_)_2_CH-, (CH_3_)_2_CHCH_2_-, cyclohexyl and C_6_H_5_CH_2_- fragments, and enrichment of the chemical space they were in was assessed (Scheme 21). Some of the prepared compounds were found to be a new type of Aurora kinase inhibitors [45].

**Scheme 22:**
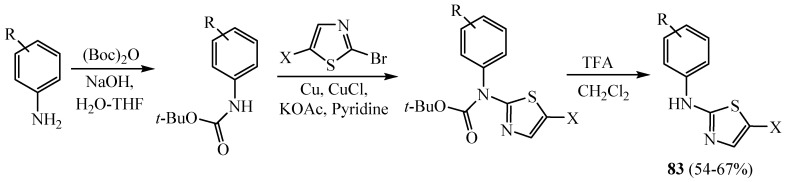
Synthesis of derivative **83.**

**Figure 1:**
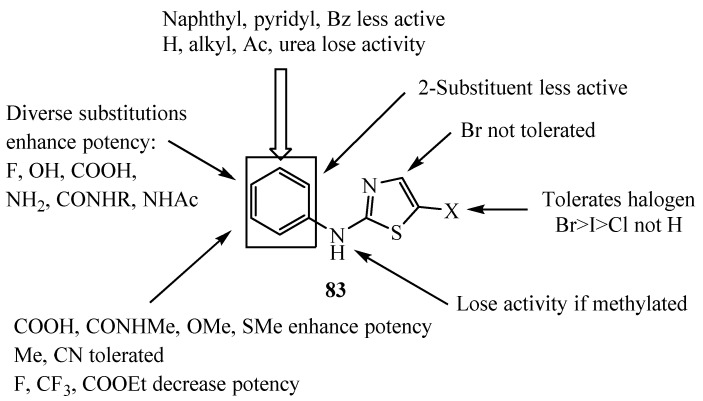
SAR of aminothiazole **83**.

Regarding the important role of Aurora family kinases which regulate events during mitosis including centrosome maturation and chromosome segregation, the misregulation of Aurora kinases due to genetic amplification and protein overexpression results in aneuploidy and may contribute to tumorigenesis. A series of 2-aminophenyl-5-halothiazoles **83** was synthesized from the reaction of 2,5 substituted thiazoles with *tert*-butyl phenylcarbamate (Scheme 22) [46,47]. The synthesized derivatives displayed different activities on Aurora kinase inhibition, with decreased histone H3 serine 10 phosphorylation. To summarize SAR for aminothiazole Aurora inhibitors, arrows indicate the position and nature of each substitution tested in a biochemical Aurora A kinase assay (Figure 1).

**Scheme 23:**
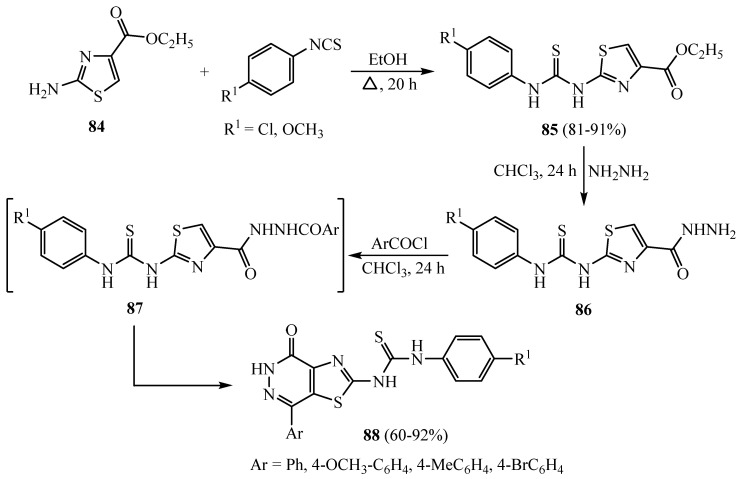
Synthesis of derivative **88.**

A series of thiazole and thiazolopyridazines, both containing the 2-thioureido function, were evaluated for in vitro antitumor activity against a cancer cell line collection. 1-(4-chloro-phenyl)-3-[4-oxo-7-(4-bromo-phenyl)-4,5-dihydrothiazolo[4,5-*d*]pyridazin-2-yl]thiourea derivative **88** (R^1^ = Cl, Ar = 4-BrC_6_H_4_) proved lethal to the HS 578T cancer breast cell line with an IC_50_ value of 0.8 µM. The title thiourea derivatives **85** were synthesized by reaction of ethyl 2-aminothiazole-4-carboxylate **84** with phenyl isothiocyanate derivatives. Then, the functional esters were reacted with NH_2_NH_2_ to give the acid hydrazides **86**, which were treated with the benzoyl chlorides to afford the corresponding 3-phenylthioureas **87**. An in situ cyclization was carried out on compound **87** to afford the thiazolo[4,5-*d*]pyridazin-2-yl]thiourea derivatives **88** (Scheme 23) [48]. The derivative **88** which contains (R^1^= Cl; Ar = 4-BrC_6_H_4_) proved to be the most active DHFR inhibitor with an IC_50_ of 0.06 µM and showed 31.4, 25.2, 37.7, 25.1 and 41.0 GI% against NCI-H522 non-small cell lung, HT29 colon, SK-OV-3 ovarian, MCF7 breast and T-47D breast cancers, respectively. Meanwhile, derivatives of compound **88** that contain (R^1^ = Cl, OCH_3_; Ar= OCH_3_, OCH_3_) were active with an IC_50_ of 0.1 and 2.5 µM, respectively. In addition, derivatives of compound **88** that contain (R^1^ = OCH_3_; Ar = Ph) showed antitumor activity against NCI-H522 non-small cell lung, HT29 colon and TK-10 renal with GI values of 31.7, 29.4 and 34.7%, respectively.

### 2.2. 2-Aminothiazoles as Antioxidant Agents

**Scheme 24:**
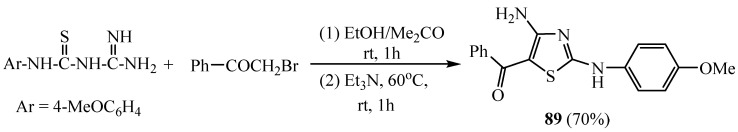
Synthesis of derivative **89.**

The energy production to fuel biological processes by oxidation is important to many living organisms. The outcomes indicated that, the synthesized derivative 4-amino-5-benzoyl-2-(4-methoxyphenyl-amino)thiazole **(89)** (Scheme 24) pointed to an important antioxidant potential in terms of scavenging free radicals. In addition, 4-aminothiazole hybrid **89** was an effective radio protector against radiation-induced damage in the liver of mice. In addition, 4-aminothiazole scaffold **89** can protect the mouse myocardium against damage and one of the possible reasons behind this protective effect can be attributed to its antioxidant property [49,50,51].

**Scheme 25:**
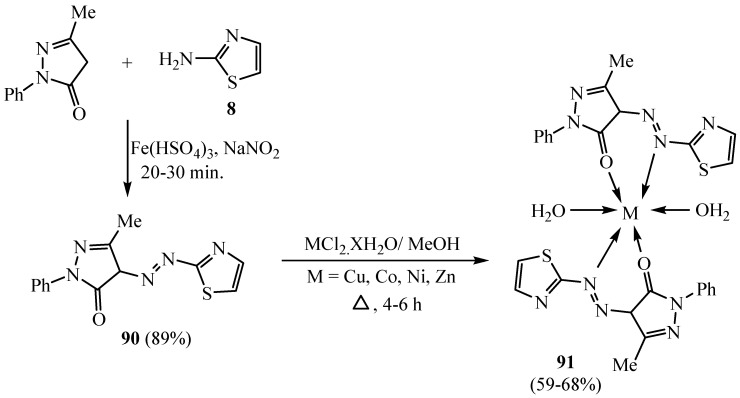
Synthesis of derivative **91.**

The 4-(thiazol-2-yl-azo)-2,4-dihydro-3*H*-pyrazol-3-one scaffold **91** was blended by a diazo-coupling reaction of 3-methyl-1-phenyl-5-pyrazolone with 2-aminothiazole **(8)** and ferric hydrogen sulfate (Scheme 25). The synthesized thiazole scaffolds **90** and **91** were assessed for an antioxidant effect, and among them, Cu(II) Co(II) and Ni(II) complexes indicated good activity in DPPH and nitric oxide scavenging [52].

**Scheme 26:**
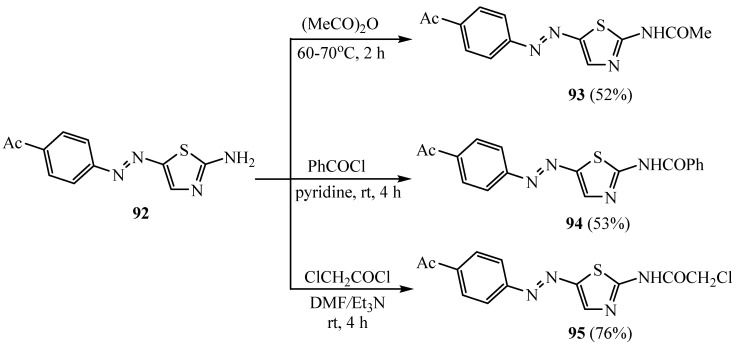
Synthesis of derivatives **93–95.**

A reactivity study of both the aryl substituent and amino group of a novel synthesized 2-amino-5-(4-acetylphenylazo)-thiazole compound and its scaffolds via different electrophilic reagents was conducted. They were biologically evaluated in vitro and in vivo for their toxicity and antioxidant activity based on liver function enzymes. The new 2-amino-5-(4-acetylphenylazo)-thiazole **(92)** was reacted with various active carbonyl reagents (Scheme 26) [53]. A convenient acetylation reaction of **92** by solvent-free acetylation with acetic anhydride afforded the *N*-acetylated product **93**. The electrophilic attack of the benzoyl cation towards **92** yielded the benzoyl amino derivative **94**. A further reaction of the highly activated chloroacetyl chloride reagent with **92** under basic conditions was performed to produce the chloroacetyl amino derivative **95** [53].

**Scheme 27:**
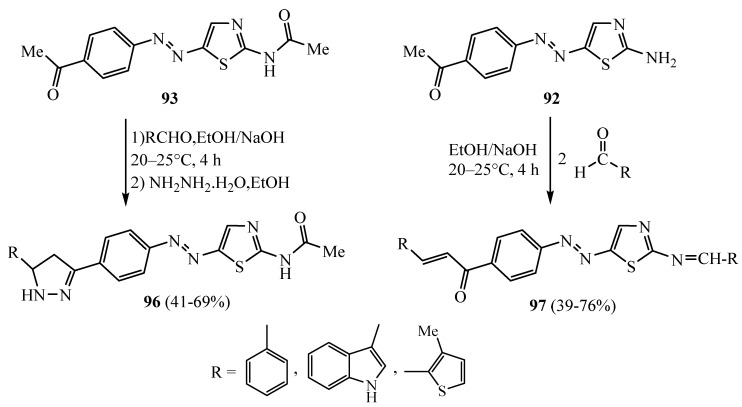
Synthesis of derivatives **96** and **97.**

Claisen–Schmidt condensation of 2-acetylamino-5-(4-acetyl-phenylazo)-thiazole **93** with an equimolar ratio of aromatic and/or heterocyclic aldehydes in sodium hydroxide and water/ethanol medium led to the formation of chalcones which reacted with hydrazine hydrate in the presence of ethanol to afford **96** (Scheme 27). The reaction of 5-arylazo-2-aminothiazole **92** with the appropriate aldehydes (two moles) under the same reaction conditions led to the formation of the chalcone-imine derivatives **97** [53]. The synthesized derivatives **96** (R = 3-methylthiopnene) and **97** (R = indole) showed a significant increase in antioxidant enzyme activities in the treated rat groups at doses of 50 and 100 mg/kg.

**Scheme 28:**
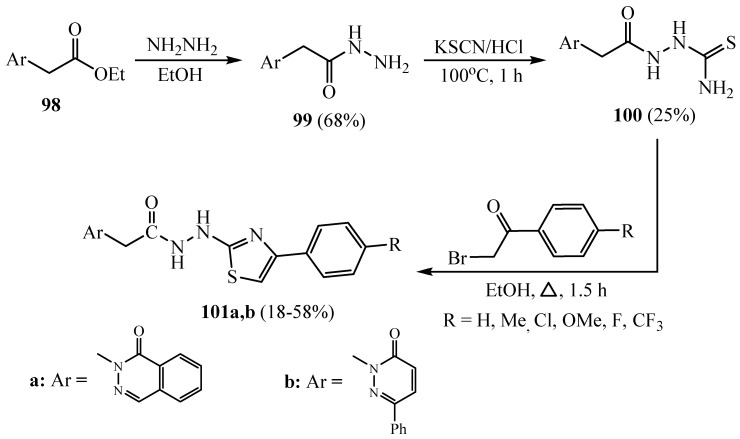
Synthesis of derivatives **99–101.**

The treatment of phthalazin-1(2*H*)-one and 6-phenylpyridazin-3(2*H*)-one compounds with ethyl bromoacetate produced the ester derivatives **98**. The reactions of compounds **98** with hydrazine hydrate afforded the hydrazide derivatives **99a** and **99b**. Then, compounds **100** hydrazine carbothioamide moieties were prepared by the reaction of compound **99** with potassium thiocyanate in the presence of hydrochloric acid (Scheme 28). Finally, hydrazinothiazoles **101** were blended by cyclization key intermediates **100** with suitable phenacyl bromides in ethanol [54]. Although the synthesized derivatives **101a,b** demonstrated good antioxidant activity, particularly in the DPPH radical scavenging assay, their inhibition activity on cholinesterase enzymes suggested a structure-specific interaction.

### 2.3. 2-Aminothiazoles as Antimicrobial Agents

**Scheme 29:**
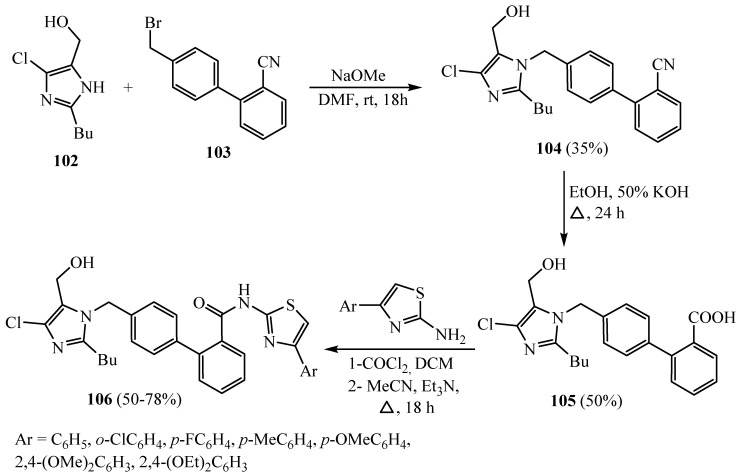
Synthesis of derivative **106.**

Thiazole and imidazole scaffolds are an essential kind of heterocyclic compound. They occupy a significant position in medicinal chemistry, showing a wide range of bioactivities. A series of 4-(2-*N*-butyl-4-chloro-5-hydroxymethyl-imidazol-1-yl-methyl-biphenyl-2 carboxylic acid-(substitutedphenyl-thiazole)-amides **106** was prepared as outlined in Scheme 29 by the conversion of the carboxylic acid derivative **105** into its acid chloride followed by acylation with many 2-aminothiazoles. The newly synthesized title compounds were screened for their in vitro antibacterial activity against S. Aureus and B. Subtilis and also for an in vitro antifungal effect against *C. Albicans* and *Aspergillus niger*. Some of the compounds exhibited encouraging outcomes [55].

**Scheme 30:**
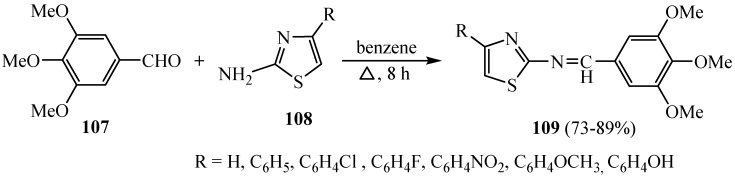
Synthesis of derivative **109.**

The condensation of 3,4,5-trimethoxybenzaldehyde **107** with 2-aminothiazole and/or 2-amino-4-(*p*-substituted/unsubstituted)-phenyl thiazole **108** (Scheme 30) was reported to furnish the corresponding Schiff bases **109**. The effect of three methoxy groups in the carbon phenyl nucleus on the course of reactions with the substituted thiazole nucleus and the compounds containing a nitro group and fluoro at para positions exhibited very good activity against both the strains, i.e., the electron-withdrawing group showed maximum inhibition in both the strains on the antibacterial and antifungal activities of the synthesized products [56].

**Scheme 31:**
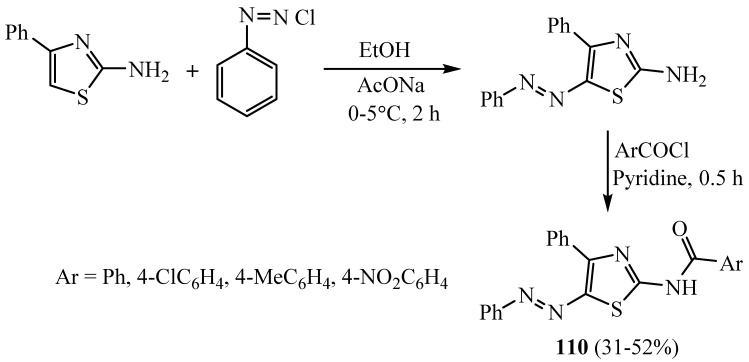
Synthesis of derivative **110.**

Some derivatives of 2-aminothiazole bearing arylazo moiety at the fifth position have been used for antimicrobial activities. The amide of 2-amino-4-phenyl-5-phenylazothiazoles derivatives **110** (Scheme 31) was obtained when 2-amino-4-phenyl-5-phenylazothiazole was acylated with appropriate substituted aromatic acid chlorides by employing the Schotten–Bauman synthesis protocol. All the synthesized compounds showed good antimicrobial activity against *E. coli*, *S. aureus*, *A. niger* and *A. oryzaeto* [57].

**Scheme 32:**
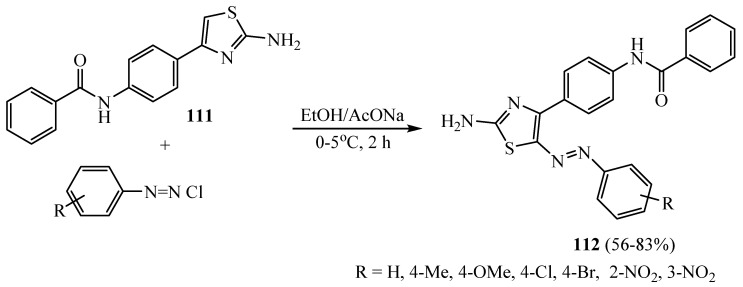
Synthesis of derivative **112.**

Coupling of diazonium salts with 2-aminothiazole derivative **111** provided the phenylazo-thiazole derivatives **112** in excellent yield (Scheme 32). The synthesized series of benzamide-linked 2-aminothiazole-based compounds showed excellent antibacterial activity and antifungal activity [58].

**Scheme 33:**
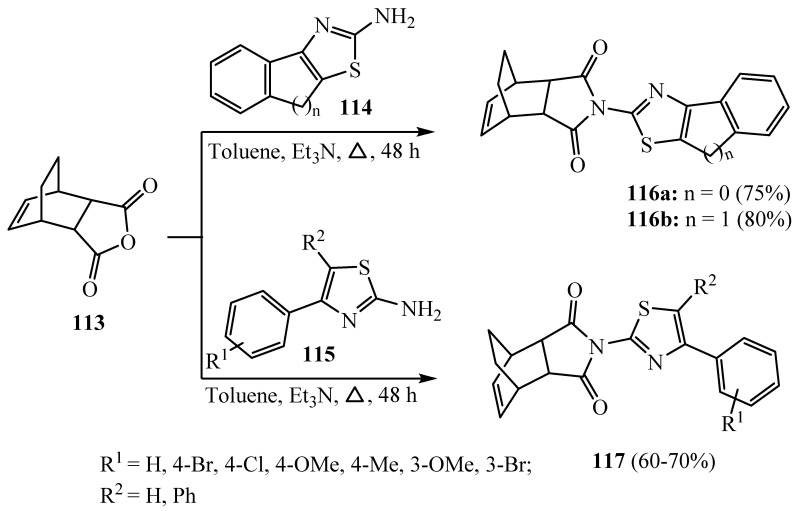
Synthesis of derivatives 116 and 117.

Ethanoisobenzofuran-1,3-dione (**113**) was obtained by the addition of maleic anhydride to cyclohexadiene. The reaction of 2-aminothiazole derivatives **114** and **115** with the anhydride derivative **113** gave a group of new 2-(4-arylthiazol-2-yl)-3a,4,7,7a-tetrahydro-1*H*-4,7- ethanoisoindole1,3(2*H*)-dione derivatives **116** and **117** (Scheme 33). According to MIC values, derivatives **117** (R^1^ = OCH_3_) and **117** (R^1^ = CH_3_) presented remarkable efficacy toward *E. coli*. Derivative **117** (R^1^ =H, R^2^ = Ph) showed significant efficacy toward P. *Aeruginosa*. Derivatives **117** (R^1^ = 4-Br), **116** (n = 0) and 117 (R^1^ = 4-Cl) displayed low activity, and **117** (R^1^ = OCH_3_) and **117** (R^1^ = CH_3_) showed remarkable efficacy toward *S. marcescens*. In summary, the utmost active derivatives are **117** (R^1^ = 4-Cl) (MIC: 0.039 μg/mL) toward C. *perfringes* and **117** (R^1^ = H) (MIC: 0.078 μg/mL) toward *A. tumefacens*. Regarding SAR, derivatives **117** containing 4-Br and 4-Cl groups were established to be the utmost active compounds according to the inhibition zone. They displayed particularly high efficacy toward the utmost utilized microorganisms [59].

**Scheme 34:**
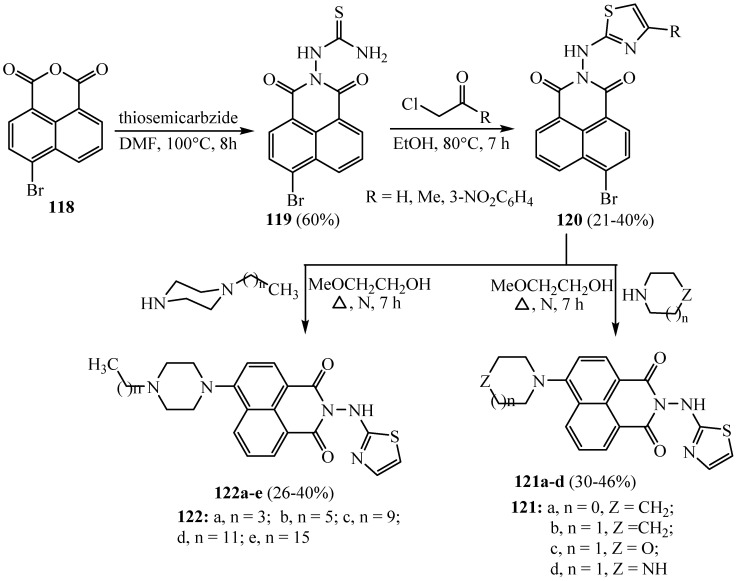
Synthesis of derivatives **119–117.**

The target naphthalimide aminothiazoles **121a–d** and **122a–e** were synthesized through multi-step reactions beginning from 4-bromo-1,8-naphthalic anhydride **118** according to Scheme 34. Condensation of **118** and thiosemicarbazide gave compound **119**, which was further cyclized with the α-halogenated carbonyl compounds to afford aminothiazole scaffolds **120**. Compound **120** was further treated with alicyclic amines to give **121a-d**. The *N*-alkylation of piperazine with alkylhalides generated mono-substituted alkyl piperazines **122a**−**e**. Piperazinyl derivatives effectively prevent the growth of methicillin-resistant *S. Aureus* and *E. coli* with MIC values of 4 and 8 μg/mL, respectively. The utmost active derivative **121d** with the NH free piperazine moiety (MIC. values from 2 to 128 micromolar) displayed the most toxicity toward Gram-positive bacteria such as *S. aureus* 29213 and *aureus* 25923 and was also effective in inhibiting Gram-negative bacteria such as *E. coli*, *E. coli* 25922, *P. aeruginosa* and *P. aeruginosa* 27853 at low concentrations. These designated 121d had massive potentiality to be more effective broad-spectrum antimicrobial agents. In addition, the extents of alkyl chains possess diverse effects on biological efficacy as in derivative **122b** with the hexyl group, which provided enhanced antibacterial efficacy in contrast to further alkyl derivatives. Likewise, when the alkyl substituents were lengthy to decyl, dodecyl and hexadecyl groups, derivatives **122c–e** showed weak activity in preventing the growth of the examined bacteria. This real idea presented that only an appropriate alkyl length chain in the piperazine ring was essential for a respectable antibacterial efficacy [8].

**Scheme 35:**
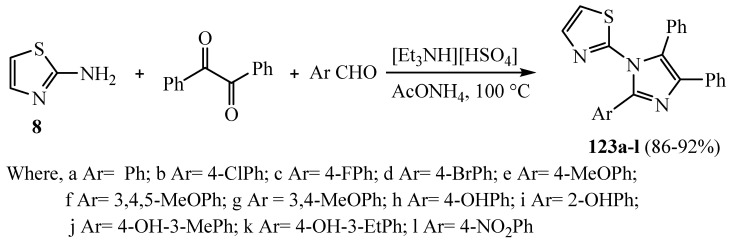
Synthesis of derivative **123a–l.**

A group of imidazole-thiazole derivatives **123a–l** were blended using the green protocol (Scheme 35). The synthesized derivatives **123a–l** were assessed for their in vitro antifungal activity, and the compounds **123j** and **123k** inhibited ergosterol biosynthesis by inhibiting enzyme cytochrome P450 lanosterol 14α-demethylase of *C. albicans*. The obtained results suggest that these compounds might inhibit fungal lanosterol 14α-demethylase related to the accepted mechanism of fluconazole [60].

**Scheme 36:**
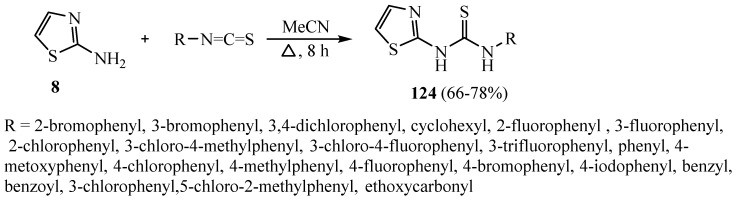
Synthesis of derivative **124.**

A series of thiazolyl-thiourea derivatives **124** was synthesized by the addition reaction of 2-aminothiazole to isothiocyanate (Scheme 36). The obtained thioureas were examined in vitro against a number of microorganisms. Initial antibacterial investigations found that halogen derivative of thiourea **124** has (3,4-dichlorophenyl) and **124** has (3-chloro-4-fluorophenyl), which reveals the supreme promising efficacy toward *staphylococcal* species. Generally, MIC results of *S. aureus* and *S. epidermidis* were displayed at 16 to 4 μg/mL. These thiourea analogues were investigated to explain their ability to prevent the formation of biofilms of eight methicillin-resistant strains of *S. epidermidis* (MRSE) [6].

**Scheme 37:**
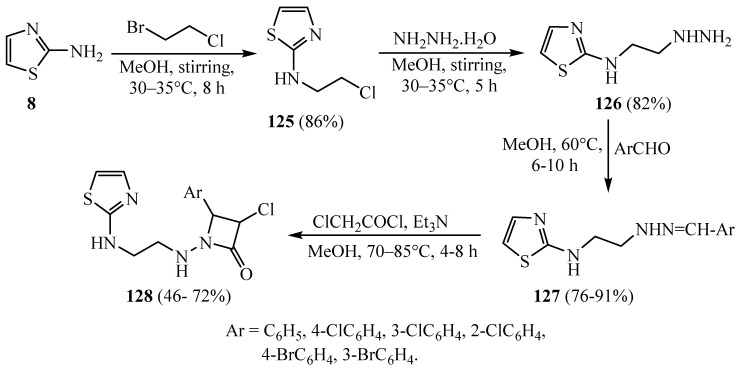
Synthesis of derivative **128.**

Chloro-4-(substitutedphenyl)-1--2-azetidinone compounds **128** (Scheme 37) were synthesized in four dissimilar steps. 2-Aminothiazole **8** on reaction with Cl(CH_2_)_2_Br at room temperature gave 2-[(2-chloroethyl) amino]thiazole **125**. Compound **125** on reaction with hydrazine hydrate at room temperature produced *N*-(2-hydrazinylethyl)-2-thiazolamine **126**. Compound **126** on further reaction with several chosen substituted aromatic aldehydes yielded substituted benzaldehyde, 2-[2-(thiazolylamino)–ethyl]–hydrazone compounds **127**. Compounds **127** on treatment with ClCH_2_COCl in the presence of Et_3_N furnished compounds **128**. The antimicrobial and antitubercular activity of the newly synthesized compounds bearing a 2-azetidinone moiety exposed that all the evaluated compounds showed moderate to good antibacterial, antifungal and antitubercular activities against the chosen microbial strains [61].

**Scheme 38:**
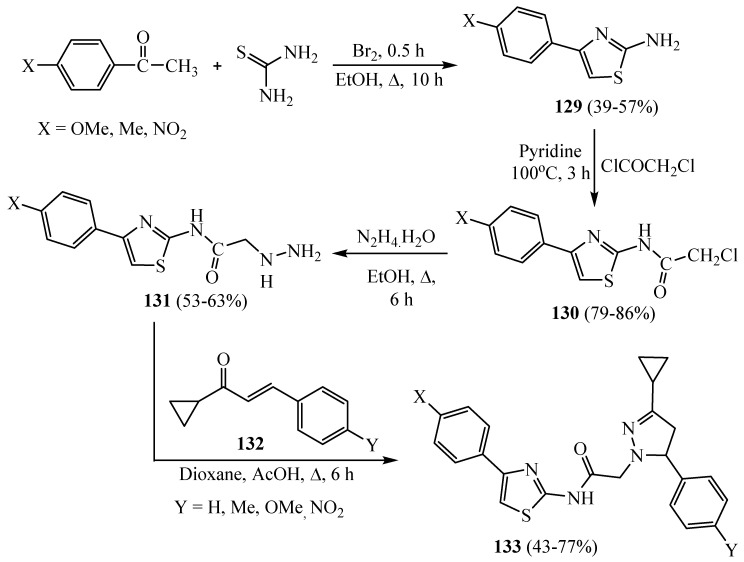
Synthesis of derivative **133.**

Some pyrazolone-linked thiazole derivatives **133** containing substituents at 1,3,5-positions were synthesized according to Scheme 38. The commencing chalcones **132** were made by conventional Claisen–Schmidt condensation by reacting suitably substituted benzaldehydes and cyclopropyl-methyl ketone. 2-Aminothiazoles **129** were gained by cyclocondensation of suitably substituted acetophenones with thiourea in the presence of bromine. Chloroacetamides **130** was obtained by reacting 2-aminothiazoles **129** with chloroacetyl chloride in the presence of pyridine. When chloroacetamides **130** were heated with hydrazine hydrate in ethanol, hydrazines **131** were obtained. When chalcones **132** were heated with hydrazines **131** in dioxane containing a few drops of acetic acid, pyrazoline derivatives **133** were gained. The target compounds **133** indicated more significant antimicrobial activity than some known standard drugs, and most compounds pointed out a moderate degree of potent antimicrobial activity [62].

**Scheme 39:**
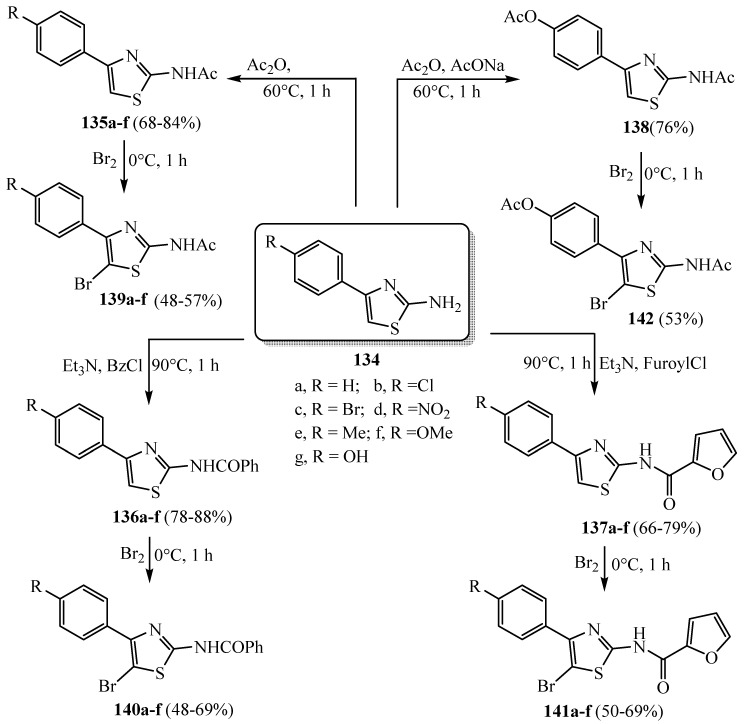
Synthesis of derivatives **135–141.**

Seven 2-amino-4-arylthiazole scaffolds **134a–g** were synthesized under microwave irradiation. Compounds **134a–f** were reacted with (CH_3_CO)_2_O, C_6_H_5_COCl and 2-furoyl chloride, respectively, to furnish thiazoles **135a–f**, **136a–f** and **137a–f** (Scheme 39). The reaction of **134g** with (CH_3_CO)_2_O led to the diacetyl derivative **139**. The bromine derivatives **139a–f**, **140a–f**, **141a–f** and **142** were obtained by the reaction of 2-amino-4-arylthiazoles, *N*-(4-arylthiazol-2-yl)-acetamides, *N*-(4-arylthiazol-2-yl)- benzamide, furan-2-carboxylic acid (4-aryl-thiazol-2-yl)-amide and acetic acid 4-(2-acetylamino- thiazol-4-yl)-phenyl ester with molecular bromine under acid conditions [63]. The synthesized compounds displayed a remarkable anti-giardial activity.

**Scheme 40:**
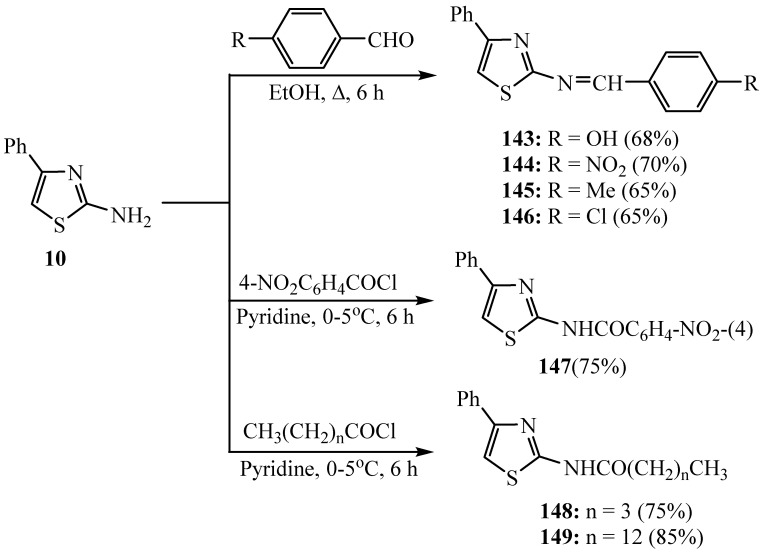
Synthesis of 2-arylideneamino-4-phenylthiazoles **143–146.**

Refluxing of compound 2-amino-4-phenylthiazole with different aromatic aldehydes in ethanol produced the corresponding 2-arylideneamino-4-phenylthiazoles **143–146** (Scheme 40) in good yields. Acylation of 2-aminothiazole **10** with various acyl halides in dry pyridine produced the corresponding amides **147–149** (Scheme 40) in high yields. Amongst the synthesized compounds investigated for the antibacterial activity, compound **144** indicated the highest activity against B. cereus. Some of the compounds pointed out low antimicrobial activities and some were incompetent to demonstrate inhibition. For the antifungal activity, all compounds pointed to outstanding outcomes against *C. Lunata* [64].

**Scheme 41:**
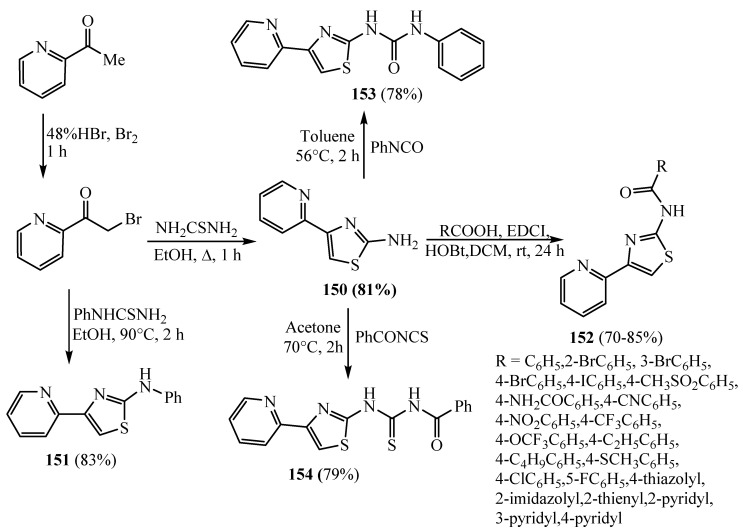
Synthesis of derivatives **150–154.**

A set of compounds was prepared from the 2-amino-4-(2-pyridyl) thiazole derivative **150** which was been synthesized by α-bromination of 2-acetylpyridine followed by condensation with thiourea. In the presence of mono-substituted carboxylic acids, 2-aminothiazole **150** underwent an EDCI (1-ethyl-3-(3-dimethylaminopropyl) carbodiimide)-mediated coupling to deliver the target amides **152**. Compound **151** was gained via the reaction of 2-bromoacetylpyridine with phenylthiourea (Scheme 41). Compounds 153 and 154 were obtained from the reaction of compound 150 with phenyl isocyanate and benzoyl isothiocyanate, respectively [65]. The antimycobacterial efficacy results for the synthesized derivatives revealed that derivative **152** with a phenyl ring which had an amide linker at position 2 had superior antimycobacterial efficacy that matched derivatives **151**, **153** and **154** which had amino, urea and acylthiourea linkers, respectively. However, derivatives **152** with a thiazole, imidazole and 2-pyridyl ring, respectively, displayed no activities toward Mycobacterium tuberculosis (M.tb). Meanwhile, analogues **152** with thiophene, 3-pyridyl, 4-pyridyl and the monosubstitution in the four position with 4-Br, 4-I, 4-CH_3_SO_2_, 4-NH_2_CO, 4-CN, 4-NO_2_ and 4-CF_3_, respectively, enhanced the activity like the unsubstituted phenyl derivative. The position of the substitution on the phenyl had an influence on activity as demonstrated by the bromo-substituted compounds with activity of the *para* > *meta* > *ortho*. Switching the 2-pyridyl substituent by a 3-pyridyl or 4-pyridyl resulted in loss of antimycobacterial activity.

**Scheme 42:**
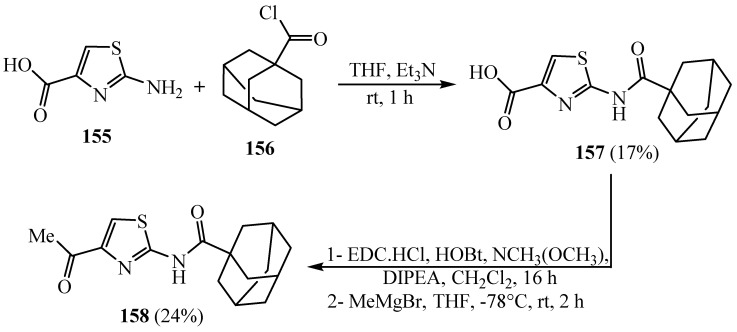
Synthesis of derivative **158.**

Treatment of the available compound 2-aminothiazole-4-carboxylate **155** with 1-adamantanoyl chloride **156** in hot tetrahydrofuran followed by conversion to Weinreb amide and Grignard in the existence of methyl magnesium bromide afforded **158** (Scheme 42) [66]. Meanwhile, the substituted amino group in C-2 position of the thiazole can accommodate a range of lipophilic substitutions, while the thiazole moiety is sensitive to modification. The synthesized derivative **158** showed respectable activity against (M.tb) growth with sub-micromolar minimum inhibitory concentrations being achieved. A demonstrative hybrid was selective for mycobacterial species over other bacteria and was rapidly bactericidal against replicating (M.tb). It was concluded that these derivatives have potential for additional progress as novel antitubercular agents.

**Scheme 43:**
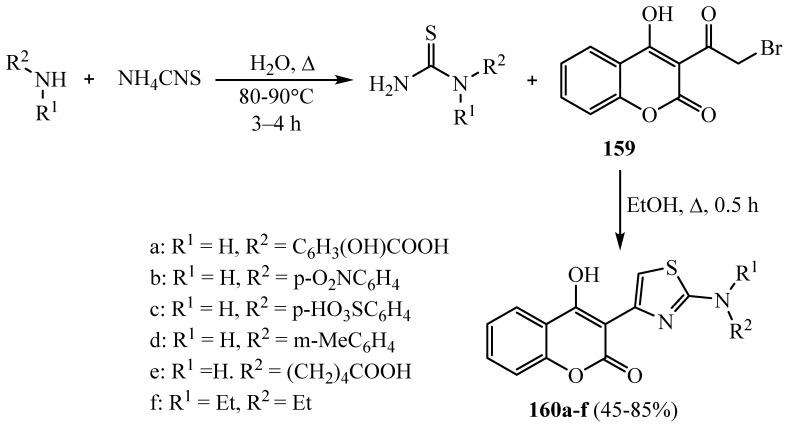
Synthesis of derivative **160a–f.**

Synthesis of 2-aminothiazole derivatives **160a-f** substituted with 4-hydroxy-chromene-2-one at the position number 4 was reported from cyclization of 3-(2-bromoacetyl)-4-hydroxy- chromene-2-one **159** with the corresponding thiourea derivatives (Scheme 43). All synthesized compounds exhibited antibacterial and antifungal activity [67].

**Scheme 44:**
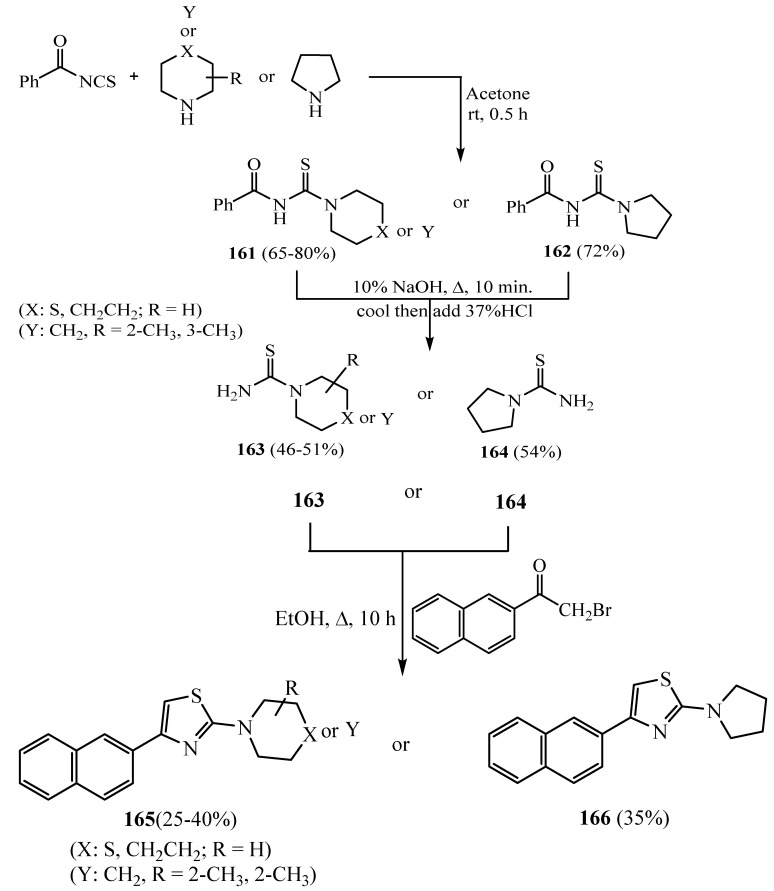
Synthesis of derivatives **161–166.**

The synthesis of new thiazole derivatives **165** and **166** from dialkyl aminothiocarbamides **163** and **164** with 2-bromo-(naphthalene-2-yl)ethanone) (Scheme 44) was considered and their in vitro antimicrobial and anticancer activity was tested. The antimicrobial properties of these naphthylthiazolylamine compounds were evaluated against various selected bacterial and fungal strains using the minimum inhibitory concentration (MIC) method. In addition, cytotoxicity studies were also carried out in Hep-G2 and A549 cell lines to examine the ability of these compounds to inhibit cell growth [68].

**Scheme 45:**
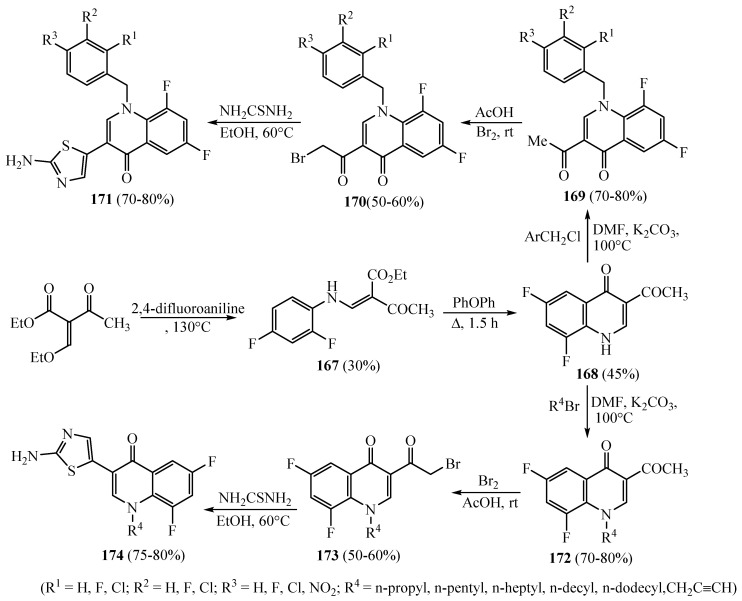
Synthesis of derivatives **167–174.**

The 2-aminothiazolyl quinolones **171** and **174** were synthesized (Scheme 45) via multi-step reactions. Ethyl (ethoxymethylene)-3-oxobutanoate was treated with 2,4-difluoroaniline to furnish ethyl 2-((2,4-difluorophenylamino)methylene)-3-oxobutanoate **(167)**, which was then further recyclized in hot phenoxy-benzene to afford the needed 3-acetyl quinolone **168**. Compound **168** was *N*-aralkylated or alkylated to afford *N*-aralkyl quinolones **169** and alkyl derivatives **172**, which were then brominated to produce the corresponding 3-(2-bromoacetyl)-quinolone derivatives **170** and **174**. The cyclization of the bromoacetyl group at the C-3 position of **170** and **173** with thiourea in ethyl alcohol at 60 °C yielded aralkyl 2-aminothiazolyl quinolones **171** (R^1^ = H, F, Cl; R^2^ = H, F, Cl; R^3^ = H, F, Cl, NO_2_) and alkyl derivatives **174** (R^4^ = *n*-propyl, *n*-pentyl, *n*-heptyl, *n*-decyl, *n*-dodecyl, CH_2_ C≡CH). The new 2-aminothiazolyl quinolones’ in vitro antimicrobial activity could effectively restrain the growth of some tested strains [69]. Antibacterial screening of the synthesized hybrids exhibited that N-1 propargyl modified 2- aminothiazolyl quinolone **174**, which presented high antibacterial activities in contrast to the rest of the derivatives against *B. typhi*. Further, this derivative exhibited equal or better activity in contrast to the two reference drugs toward *S. dysenteriae* and *P. aeruginosa*. Likewise, it was found that a shorter carbon chain such as the propyl derivative was more favorable in exerting antibacterial efficacy in comparison to norfloxacin and chloromycin as standard drugs. However, in the pentyl, octyl, decyl and dodecyl chains, a decrease in antibacterial efficacy was observed. Meanwhile, the monoflouroderivatives **171** were more active than the monochloro-derivatives **171**. Mainly, the derivative **171** with the substituent para chloro on the phenyl ring could prevent the growth of *S. dysenteriae* (MIC = 4 mg/mL). Amazingly, the activities of derivative **171** with the electro-donating OCH3 group and **171** with the electro-withdrawing NO_2_ group were not greatly diverse alongside the utmost strains and both presented comparably weak bioactivity.

**Scheme 46:**
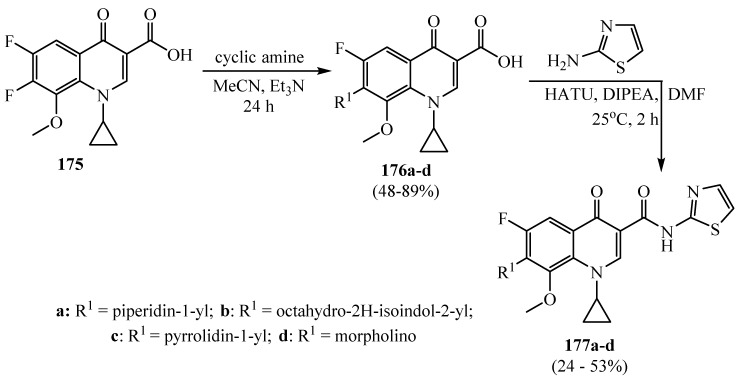
Synthesis of derivative **177a–d.**

The synthesis of *N*-thiazolyl amide fluoroquinolone derivatives **177a**-d involved the reaction sequence of nucleophilic aromatic substitution followed by acid derivatization to amides (Scheme 46). Amino-substituted fluoroquinolone compounds **176a-d** were gained by heating 1,4-dihydroquinoline-3-carboxylic acids **175** with cyclic amine in acetonitrile and triethyl amine [70]. Further, the prepared derivatives were used to investigate non-carboxylic acid fluoroquinolones with an objective to enhance the anti-staphylococcal activity and improve their toxicity profile.

**Scheme 47:**
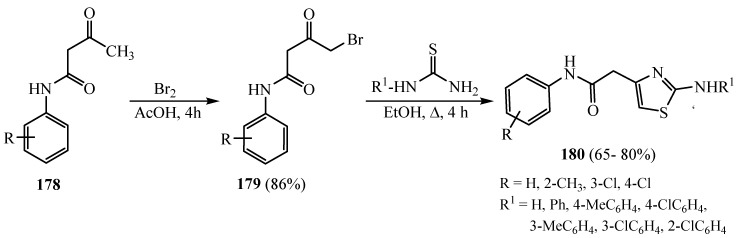
Synthesis of derivative **180.**

From ɷ-bromoacetoacetanilides **149** and thiourea/phenyl thioureas, a series of 4-arylacetamido-2-amino- and 2-arylamino-1,3-thiazoles **180** was synthesized (Scheme 47). The compounds were assessed for their in vitro antibacterial, antifungal and antioxidant activities [71].

### 2.4. 2-Aminothiazoles as Anti-Inflammatory Agents

**Scheme 48:**
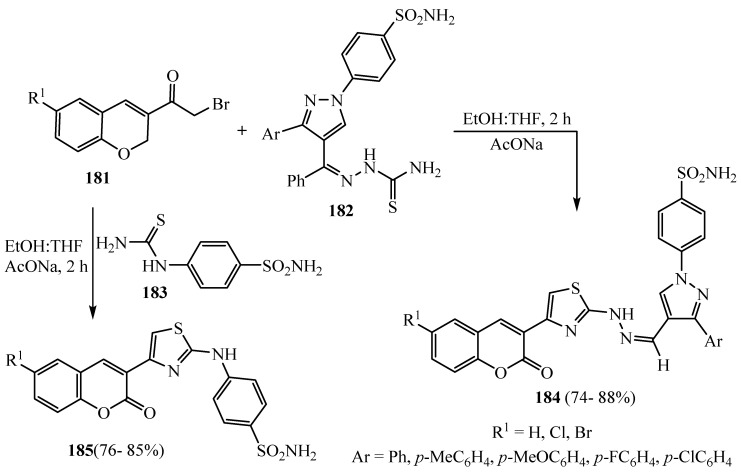
Synthesis of derivatives **184** and **185.**

The synthetic pathway used to synthesize the target thiazolyl-hydrazinomethylidene pyrazoles **184** and *N*-substituted anilinothiazoles **185** are described in (Scheme 48). The present synthesis of thiazolyl-hydrazinomethylidene pyrazoles **184** makes up the condensation of appropriate 6-substituted-3-bromoacetylcoumarin **181** with suitable pyrazole-4-carbaldehyde thiosemicarbazone **182** in the presence of sodium acetate. 4-Thioureido-benzenesulfonamide **183** was treated with different 3-bromoacetylcoumarin compounds **182** in a hot mixture of ethyl alcohol and tetrahydrofuran in the presence of CH_3_COONa to give *N*-substituted anilinothiazole derivatives **185**. All the synthesized thiazolyl-hydrazinomethylidene pyrazoles **184** and N-substituted anilinothiazoles **185** were assessed for there in vivo anti-inflammatory activity [72].

**Scheme 49:**
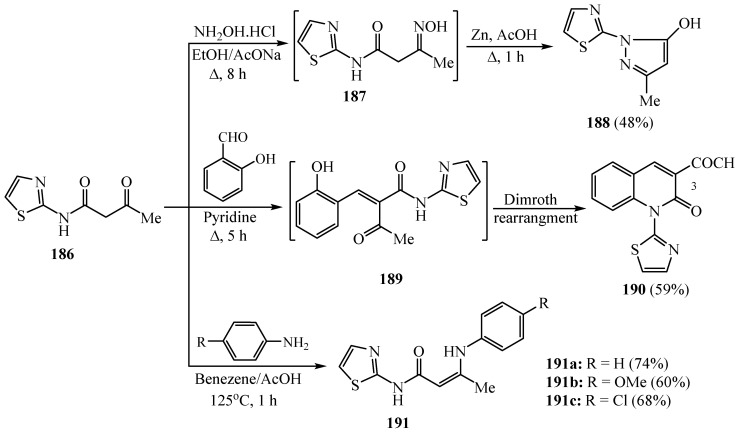
Synthesis of derivatives **188–191.**

A series of thiazolyl derivatives **188**, **190** and **191** was synthesized from the reactions of 3-oxo-*N*-(thiazol-2-yl)butanamide **186** with hydroxylamine, salicylaldehyde and aromatic aldehyde derivatives through the next synthetic pathway as shown in Scheme 49. The synthesized derivatives displayed inhibitory activities toward both the COX-1 isozyme (IC_50_ = 1.00–6.34 μM range) and the COX-2 isozyme (IC_50_ = 0.09–0.71 μM range), with COX-2 selectivity indexes in the range of 3.03 to 16 in comparison with the COX-2 selective standard drug celecoxib (COX-1, IC_50_ = 7.21 μM, COX-2, IC_50_ = 0.83 μM and S.I. = 8.68) [73].

**Scheme 50:**
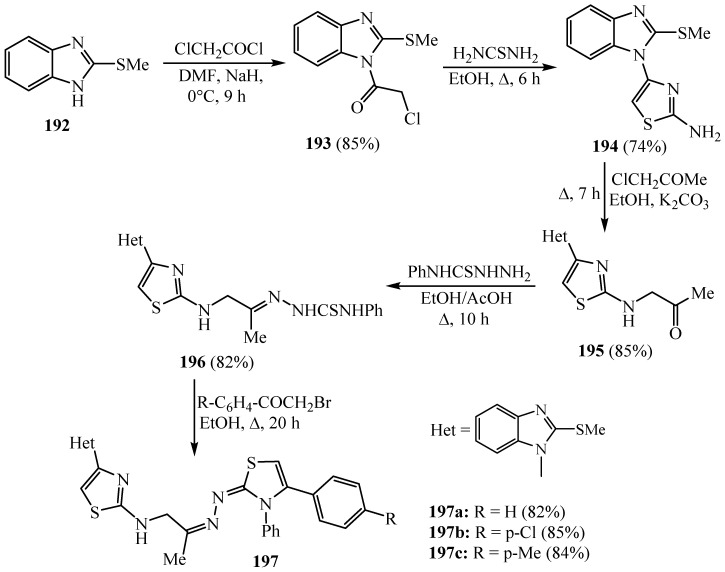
Synthesis of derivative **197a–c.**

Recently, a new series of 2-aminothiazoles bonded with 2-methylthiobenzimidazole was prepared to investigate their anti-inflammatory properties on cyclooxygenase (COX) and lipoxygenase (15-LOX) enzymes’ inhibition Scheme 50. The synthesized hybrids containing the acetyl group **195**, phenyl thiosemicarbazone **196** and 1,3-thiazolines **197a-c** were demonstrated to be the most selective COX-2 as well as 15-LOX inhibitors, that is, due to the fact they provided a collaboration of not only molecular volume advantages but also steric, electronic, hydrogen bonding and hydrophobic advantages that are essential to confirm the optimal molecular interactions with the specific biological boards and to inhibit their biological responses. Currently, the importance of these derivatives connected to diverse aromatic and heterocyclic rings for evolving innovative anti-inflammatory agents with dual COX-2 /15-LOX enzyme inhibitory efficacy is avowed [74].

**Scheme 51:**
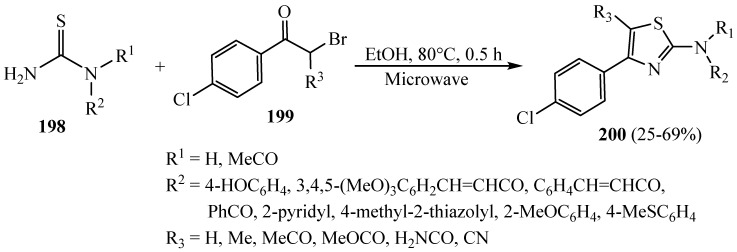
Synthesis of derivative **200.**

Finally, some new 4-(4-chlorophenyl)thiazol-2-amines **200** were prepared via cyclic condensation of an α-bromoketone **199** and N-substituted thiourea **198** in anhydrous ethanol, stirring under microwave irradiation at 80 °C for 30 min. The synthesized hybrids were examined to evaluate their inhibitory effectiveness against bovine pancreatic DNase I (Scheme 51). The in vitro evaluation of DNase I inhibition was based on spectrophotometric measurement of acid-soluble nucleotide formation at 260 nm. Inhibition of 5-LO activity was determined both in an intact cell system using freshly isolated polymorphonuclear leukocytes (PMNL) and in a cell-free assay using partially purified recombinant 5-LO, and 5- LO product formation was determined by HPLC. The synthesized hybrids reserved DNase I with IC_50_ values under 100 µM, and the derivative with (R^1^ = H, R^2^ = phenol and R^3^ = amide group) displayed a potent IC_50_ = 79.79 µM, where the crystal violet, used as a positive control in the absence of a “golden standard”, exhibited almost 5-fold weaker DNase I inhibition [75].

## 3. Conclusions

The heterocycles of 2-aminothiazole scaffolds occupy a dominant part in organic/medicinal chemistry in relation to their reactivity and biological activity and mostly act as pharmacophores. The present review summarizes the literature reports of the various synthetic routes for 2-aminothiazole-containing molecules with four different biological activities (namely, anticancer, antioxidant, antimicrobial and anti-inflammatory activities). The presented information in this review is valuable for future innovation. The simple synthesis of 2-aminothiazole hybrids bids the structure–activity revisions of several substitutions of this multilateral pharmacophore. Further, several 2-aminothiazoles and their derivatives were generally utilized as drugs in the treatment of various diseases, which has led to their extensive improvements. Attributable to their broad scale of biological activities, their skeleton variants have attracted the attention of many biologists. This review highlighted the recently synthesized 2-aminothiazole-containing compounds within the last thirteen years ago. Further, the synthetic strategies developed for the admission of the recent 2-aminothiazole derivatives (N-substituted, 3-substituted, 4-substituted, multi-substituted, aryl/alkyl substituents or acyl/other substituents) were presented. The reported literature revealed several synthetic pathways of those 2-aminothiazoles related to four different biological activities (anticancer, antioxidant, antimicrobial and anti-inflammatory activities). It is hoped that this review will be useful in displaying the rationalistic designs of 2-aminothiazole-based medical synthetic pathways.
